# Emerging frontiers of cell-free DNA fragmentomics

**DOI:** 10.20517/evcna.2022.34

**Published:** 2022-12-21

**Authors:** Xi Hu, Spencer C. Ding, Peiyong Jiang

**Affiliations:** ^1^Centre for Novostics, Hong Kong Science Park, Pak Shek Kok, New Territories, Hong Kong, China.; ^2^Li Ka Shing Institute of Health Sciences and Department of Chemical Pathology, The Chinese University of Hong Kong, Prince of Wales Hospital, Shatin, New Territories, Hong Kong, China.; ^3^State Key Laboratory of Translational Oncology, The Chinese University of Hong Kong, Shatin, New Territories, Hong Kong, China.

**Keywords:** Plasma DNA, urinary DNA, fragmentomics, pregnancy, oncology, transplantation, virology

## Abstract

Analysis of cell-free DNA (cfDNA) in the blood has shown promise for monitoring a variety of biological processes. Plasma cfDNA is a mixture comprising DNA molecules released from various bodily tissues, mediated by characteristic DNA fragmentations occurring during cell death. Fragmentation of cfDNA is non-random and contains tissue-of-origin information, which has been demonstrated in circulating fetal, tumoral, and transplanted organ-derived cfDNA molecules. Many studies have elucidated a plurality of fragmentomic markers for noninvasive prenatal, cancer, and organ transplantation assessment, such as fragment sizes, fragment ends, end motifs, and nucleosome footprints. Recently, researchers have further revealed the large population of previously unidentified long cfDNA molecules (kilobases in size) in the plasma DNA pool. This review focuses on the emerging biological properties of cfDNA, together with a discussion on its potential clinical implications.

## INTRODUCTION

Liquid biopsy supports numerous approaches that are important for noninvasive prenatal testing (NIPT)^[1-3]^ and cancer detection^[4-7]^. The use of maternal plasma DNA has enabled the rapid global adoption of NIPT for fetal chromosomal abnormalities in clinical practice, profoundly reducing unnecessary invasive tests. Continuous efforts have been extended to noninvasive cancer detection, attempting to create a similar paradigm shift in oncology^[8]^. Many studies have focused on deciphering new biological properties of cell-free DNA (cfDNA) molecules, aiming to further improve the performance of NIPT^[9]^ and achieve cfDNA-based cancer detection at the early stage of a tumor^[6]^. This review focuses on several key characteristics of cfDNA that have been recently unveiled, including the properties of short and long cfDNA molecules, fragment ends, nucleosome footprints, and topological shapes, together with discussions on its potential clinical implications.

## FRAGMENT SIZES

### Short cfDNA molecules

Lo *et al.* deciphered the characteristic fragmentations of fetal and maternal DNA molecules in the maternal plasma DNA of a pregnant woman using massively parallel sequencing technology^[10]^. There was a major peak at 166 bp in the respective fetal and maternal size profiles, with a series of 10-bp oscillations in short size ranges^[10]^, suggesting that the cfDNA molecules are possibly associated with nucleosomal structures. The size distribution of fetal cfDNA molecules displayed a secondary major peak at 143 bp, with enhanced amplitudes across the 10-bp oscillations. This observation suggests that the fetal cfDNA molecules are generally shorter than the maternal DNA molecules. Lo *et al.* speculated that the shortened size of the fetal DNA molecules might be in part attributed to the preferential trimming of approximately 20-bp linkers in the fetal genome as a result of less protection from histones^[10]^. Hence, cfDNA fragmentation is non-random and is associated with tissues of origin. Many studies could reproduce similar characteristic size profiles in plasma DNA of healthy control individuals^[6]^ and patients with organ transplantations^[11]^, different cancers^[6,12]^, and autoimmune diseases^[13]^. The liver-derived and tumor-derived cfDNA molecules were reported to be generally shorter than background DNA molecules of hematopoietic origin in patients with liver transplantation^[11]^ and various cancers^[6,12,14,15]^, respectively.

The discovery of these non-random cfDNA fragmentation patterns has promoted the emergence of novel diagnostic tools. Yu *et al.* used the principle that fetal DNA is shorter than maternal DNA molecules to develop an approach for detecting fetal chromosomal aneuploidies^[16]^. The affected chromosome in a trisomic fetus would increase the relative contribution of fetal DNA molecules to the maternal plasma DNA pool, thus further enriching short cfDNA molecules from that chromosome. The affected chromosome in a monosomic fetus, however, would relatively reduce the contribution of short DNA molecules from that chromosome and display a lengthened size. Therefore, measuring cfDNA molecules could provide information on fetal chromosomal trisomy 21 and 18. This approach could achieve 100% sensitivity and specificity, as reported in the study^[16]^, which was comparable to the count-based approach^[2]^. Importantly, the integrated analysis of fragment sizes and counts facilitated the determination of the fetal and/or maternal origin of the copy number aberrations seen in maternal plasma, thus supporting a more accurate interpretation of NIPT results^[9]^. Moreover, the researcher attempted to enable the detection of fetal *de novo* mutations in the presence of the overwhelming maternal background DNA. The positive predictive value (PPV) was reported to be extremely low^[17]^. In contrast, using the size-based bioinformatic filter together with adjusted read alignment parameters, fetal *de novo* mutations could be detected with a PPV that was two orders of magnitude higher than previously reported^[18]^.

These developments with fetal cfDNA have also encouraged researchers to actively explore the properties of tumor-derived cfDNA in plasma. Many studies have demonstrated that using size information could help achieve better performance in cancer detection. For example, Jiang *et al.* demonstrated that the PPV of detecting tumor-derived mutations in the plasma of hepatocellular carcinoma (HCC) patients could be improved by up to 85% by taking advantage of how the tumor-derived DNA is shorter than background DNA^[6]^. Using this similar analytical strategy, it was feasible to distinguish clonal hematopoiesis from tumor-derived mutations in plasma DNA^[19]^. Additionally, Mouliere reported that an enhanced detection of tumor-derived DNA molecules could be achieved by fragment analysis, for example, by employing the physical size selection of shorter DNA molecules^[12]^.

Since it became clear that the 166-bp cfDNA molecules were related to the nucleosome core with the linker DNA, while the 143-bp cfDNA molecules represented the nucleosomal core without the linker^[10]^, “nucleosomal tracks” were constructed *in silico* by maternal plasma DNA data combined from hundreds of cases^[20]^. The ratio of fragments starting within 73 bp upstream and downstream of the middle of the nucleosomal core deduced from the “nucleosomal tracks” was able to inform the fetal DNA fraction^[20]^. The fetal DNA fraction is a crucial parameter for NIPT. Such nucleosomal tracks could also be reproduced in patients with different cancers, with the use of a metric called window protection score (WPS)^[21]^. The WPS was defined as the number of molecules spanning a 120-bp genomic window minus those ending within that window^[21]^.

**Figure 1:**
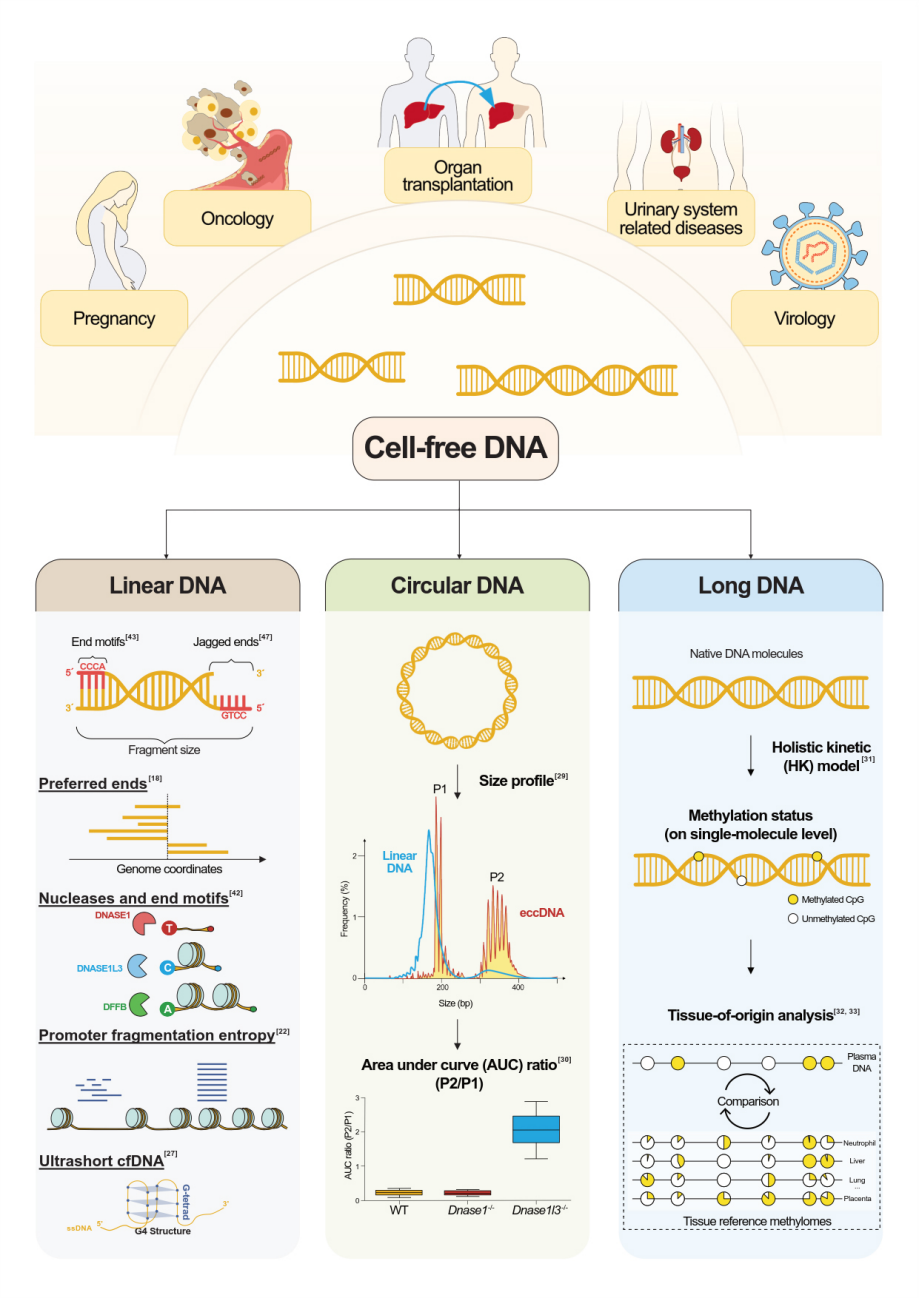
Summary of the emerging frontier of cell-free DNA and potential clinical implications. (Top) Cell-free DNA (cfDNA) is a mixture of fragments released from various healthy and diseased cells in different tissues. The fragmentation of cfDNA is non-random, bearing information directly related to the tissue of origin. In addition, cfDNA methylation can also be used to trace each cfDNA molecule"s cellular identity, showing promise for various clinical applications. The fragmentomic and epigenetic analyses of cfDNA offer the opportunity to develop novel diagnostic tools for assessing pregnancy, oncology, organ transplantation, urinary system-related diseases, and virology. (Bottom left) Non-random fragmentation of cfDNA molecules can be reflected by fragment sizes, preferred ends, end motifs, jagged ends, nuclease activities, promoter fragmentation entropy, as well as ultrashort cfDNA. (Bottom middle) Extrachromosomal circular DNA (eccDNA) has a different size profile compared with linear DNA. In human plasma DNA, linear DNA has a pronounced peak at ~166 bp. In contrast, eccDNA has two prominent peaks, the first peak (P1) is at ~200 bp and the second peak (P2) is at ~350 bp^[29]^. Interestingly, the area under curve (AUC) ratio of P2 to P1 is significantly increased in *Dnase1l3* knockout mouse (*Dnase1l3^-/-^*) plasma DNA, whereas *Dnase1^-/-^* mice do not show an apparent difference from wildtype mice (WT). This suggests that DNASE1L3 possibly prefers to cut long eccDNA^[30]^. (Bottom right) A large population of long cfDNA molecules was recently observed in plasma using single-molecule real-time sequencing. The direct methylation detection of native cfDNA molecules enables the determination of tissue of origin for each single cfDNA molecule^[31-33]^.

Esfahani *et al.* recently demonstrated that using the size variability of the cfDNA fragments surrounding the transcription start sites (TSS) helped predict the expression levels of genes^[22]^. The transcriptionally active regions tended to have decreased nucleosome occupancies, conferring more random cleavages and higher DNA fragment length variability. A metric called promoter fragmentation entropy (PFE) was used to quantify such variability in cfDNA fragments originating from promoter regions [Figure 1, Bottom left]. PFE was found to be strongly correlated with RNA expression levels. To further evaluate the clinical utility of PFE, Esfahani *et al.* used targeted deep sequencing of selected biomarker gene promoters that had tumor-specific RNA expression profiles, called “epigenetic expression inference from cell-free DNA-sequencing” (EPIC-seq)^[22]^. This method could distinguish lung carcinoma subtypes, with the area under the receiver operating characteristic curve (AUC) values of 0.91 and 0.83 in the training and validation datasets, respectively, for distinguishing individuals with non-small cell lung cancer from controls. The measurement of fragment size variability in gene promoters could provide noninvasive biomarkers for cancer detection. Notably, 90% of the patients were detected at the late stage of cancer (stage III or IV) for PFE-based EPIC-seq data analysis. Moreover, many recent studies have demonstrated that fragmentation patterns could serve as biomarkers for predicting treatment outcomes, such as recurrences of nasopharyngeal carcinoma (NPC)^[23]^, treatment outcomes of diffuse large B-cell lymphoma (DLBCL)^[24]^, and response to immunotherapy^[22]^.

The size characteristics described above were based on double-stranded DNA sequencing library preparation. Interestingly, several recent studies revealed the presence of single-stranded cfDNA molecules in maternal plasma DNA, showing the significant enrichment of short DNA molecules^[25,26]^. However, the fetal DNA fraction appeared to be similar between results generated by single-stranded and double-stranded DNA sequencing preparations^[25]^. Furthermore, some studies demonstrated that a considerable population of plasma cfDNA fragments centered at ~50 bp was present in the plasma DNA pool using modified single-stranded DNA library preparations. These included combining high-affinity magnetic bead-based DNA extraction with single-stranded DNA sequencing library preparation^[27]^ and adjusting the concentration of isopropanol to retain the low molecular weight nucleic acids for downstream analysis^[28]^. Notably, such ultrashort cfDNA fragments were found to be enriched in the accessible chromatin regions of blood cells, particularly in promoter regions that potentially harbor G-quadruplex (G4) DNA secondary structures [Figure 1, Bottom left]. G4-positive promoter chromatin accessibility was reportedly decreased in cancer patients, potentially providing another type of biomarker for cancer detection^[27]^.

### Long cfDNA molecules

In contrast to most studies focusing on short DNA molecules (< 500 bp), Yu *et al.* recently revealed the existence of long fetal DNA in maternal plasma by single-molecule real-time (SMRT) sequencing (Pacific Biosciences, PacBio)^[32]^. A substantial proportion of long fetal cfDNA molecules was detected in maternal plasma DNA, with 15.5%, 19.8%, and 32.3% in the first, second, and third trimesters of gestation, respectively. The longest fetal cfDNA was more than 23 kb in length. Yu *et al.* further elucidated that the proportion of long cfDNA molecules could serve as a biomarker for preeclampsia. Compared with the short-read sequencing technology (Illumina), the size metric deduced by SMRT sequencing showed a better differentiation between pregnancies with and without preeclampsia^[32]^. The previous short-read sequencing technologies (Illumina) were not suited for long cfDNA molecule analysis because bridge amplification on sequencing flow cells does not favor long DNA molecules^[34,35]^.

The presence of long cfDNA in plasma^[32]^ could extend the boundary of liquid biopsy from short cfDNA molecules to kilobase-long cfDNA molecules. One important implication of the presence of long cfDNA in plasma is that the use of methylation patterns across a series of CpGs in a long cfDNA molecule theoretically makes it possible to determine the tissue of origin for each plasma DNA molecule [Figure 1, Bottom right]. With the use of an artificial intelligence algorithm, Tse *et al.* demonstrated direct methylation detection of a native DNA molecule using the kinetic signals generated by a DNA polymerase during SMRT sequencing [the holistic kinetic (HK) model]^[31]^. Thus, the cell identity of each individual plasma DNA molecule could be traced by the “intrinsic molecular barcode” through the HK model. Yu *et al.* demonstrated such a concept in the maternal plasma of pregnant women, leveraging the abundance of CpG sites on long DNA molecules to identify the fetal-derived DNA molecules with an accuracy of 0.88^[32]^. Another independent study illustrated that single-molecule sequencing (PacBio) enabled long cfDNA detection and direct methylation analysis for cancer patients^[36]^. Such a proof-of-concept study showed that using long cfDNA molecules could enhance the discriminative power up to an AUC of 0.91, compared with short cfDNA with an AUC of 0.75^[36]^. Hence, a previously unidentified long cfDNA population may open many new possibilities for liquid biopsy in NIPT and cancer detection.

## FRAGMENT ENDS

### Preferred Ends

The characteristic size patterns of cfDNA from various tissues indicate the non-random cleavages occurring across genomes during DNA fragment shedding into blood circulation. From results of ultra-deep sequencing of maternal plasma DNA, it was reported that a subset of genomic coordinates over-represented cfDNA fragment ends, which are referred to as “preferred ends”^[18]^ [Figure 1, Bottom left]. The plasma DNA molecules terminating at those preferred ends exhibited tissue specificity. For example, those cfDNA molecules from fetal-preferred ends were shorter in size and correlated with fetal DNA fractions^[18]^. Additionally, preferred ends were clustered in line with nucleosomal patterns^[37]^, pervasively existing in various tissues, such as liver-specific preferred ends in the plasma of patients with liver transplantation and tumor-specific preferred ends in HCC patients^[37]^. Using tumor-preferred ends in plasma DNA could facilitate HCC detection, with an AUC of 0.88^[37]^. As a large number of preferred ends would be present in plasma DNA, the in-depth analysis of preferred ends potentially paves the way to the sensitive detection of cancer at its early stage^[37,38]^.

### End Motifs

Researchers studied the compositions for several nucleotides (denoted by “k”) proximal to the 5’ end of a cfDNA molecule, namely k-mer end motifs [Figure 1, Bottom left]. Some previous studies showed that the 5’ end motifs of plasma DNA preferentially started with “C” nucleotides^[39,40]^. However, the biological mechanism for generating plasma end motifs was still obscure in these studies. The tissue specificity and clinical utility regarding plasma DNA end motifs had not been explored at that time. Recently, Serpas *et al.* used knockout (KO) mice models to reveal that the plasma DNA size and end motifs profile were associated with DNA nucleases^[41]^. It was reported that the top six 4-mer end motifs (a total of 256 motifs), which all started with “CC” in WT mouse plasma (7.4%), significantly declined in mice with *Dnase1l3* deletion (4.2%). In contrast, no significant effect was present with *Dnase1* KO in one study^[41]^. DNASE1L3 was thus believed to be an important DNA nuclease for generating “CC” ends of plasma DNA molecules.

To determine whether the effect of DNASE1L3 is at a systemic or local tissue level, Serpas *et al.* designed one experiment using a pregnant mouse model in which *Dnase1l3^−/−^* mice (no functional copy of the *Dnase1l3* gene) were pregnant with *Dnase1l3^+/−^* fetuses (one functional copy of the *Dnase1l3* gene). A partial normalization of end motifs was observed for both maternal and fetal DNA, with a higher degree of end motif restoration for fetal DNA molecules^[41]^. Therefore, DNASE1L3 was believed to act on plasma DNA fragmentation in both a systemic and local manner. The distinct roles of DNASE1, DNASE1L3, and DNA Fragmentation Factor Subunit Beta (DFFB) on cfDNA fragmentation were further demonstrated with the use of different KO mouse models combined with various *in vitro* blood incubations^[42]^. Han *et al.* found that the generation of cfDNA molecules might involve a series of DNA nucleases that function in a stepwise manner, including extracellular and intracellular levels. Such a stepwise nuclease-mediated cfDNA fragmentation model suggested that the cfDNA was initially cleaved intracellularly with DFFB, preferentially forming A-end fragments, followed by cleavage events mediated by DNASE1L3 and DNASE1, preferentially producing C-end fragments and T-end fragments, respectively^[42]^.

Intriguingly, alterations of DNASE1L3-cutting signatures (“CC” end motifs) could be mirrored in the plasma of human subjects with DNASE1L3 deficiency. These included patients with familial monogenic systemic lupus erythematosus (SLE) with DNASE1L3 mutations and many cancers with downregulated DNASE1L3 expression. For example, the most frequently observed end motif in the plasma of healthy human subjects was CCCA, while this end motif was significantly decreased in patients with HCC, colorectal cancer, lung cancer, nasopharyngeal carcinoma, and head and neck squamous cell carcinoma^[43]^. Making full use of 256 motifs achieved an AUC value of 0.86 in differentiating patients with and without cancers. Many other groups were used to validate a number of potential clinical applications by employing plasma DNA end motifs^[38,44,45]^. Moreover, transducing *Dnase1l3* into *Dnase1l3*-deficient mice could partially restore the altered end motif profiles of *Dnase1l3*-deficient mice to the profiles of WT mice^[46]^. Taken together, the analysis of nuclease-associated cutting signatures could provide potential diagnostic tools for detecting and monitoring various diseases associated with DNA nuclease activities.

### Jagged ends

Double-stranded plasma DNA was generally subjected to end-repair steps that experimentally altered the ends, but whether single-stranded DNA exists at the ends of a double-stranded cfDNA molecule (referred to as jagged ends) has remained unknown for many years [Figure 1, Bottom left]. To address this research gap in the area of fragmentomics, Jiang *et al.* used the DNA end-repair process to introduce differential methylation signals into the complementary strand of the single-stranded jagged end^[47]^. The resulting methylation signal density in the newly generated strand reflected the quantity of jagged ends. Jiang *et al.* found that plasma DNA molecules contained a significantly higher level of jaggedness than the sonicated DNA molecules, with 88% of plasma DNA molecules carrying jagged ends. Interestingly, the property of plasma DNA jagged ends appears to be associated with tissue of origin. For instance, in pregnant women, the fetal DNA was shown to harbor higher jaggedness than background DNA molecules mainly of hematopoietic origin. A similar pattern was observed in tumor-derived DNA molecules in plasma from HCC patients^[47]^.

Ding *et al.* recently elucidated the relationship between the jagged end length and nucleosomal structures when the DNA nuclease activity of DNASE1, DFFB, or DNASE1L3 is changed^[48]^. This study concluded that DNASE1 was responsible for jagged ends across a wide size range, extending from linker DNA to the nucleosomal core. DFFB tended to generate blunt or short jagged ends in linker DNA between two nucleosomes. DNASE1L3 would play different roles in short and long cfDNA molecules. In particular, the deletion of DNASE1L3 would increase the jaggedness in fragments shorter than 150 bp, but decrease the jaggedness in fragments involving multi-nucleosomal structures. The aberration in plasma DNA jaggedness could serve as a biomarker for human subjects with SLE^[48]^.

## FRAGMENT SHAPES

In addition to the linear form of cfDNA molecules, many recent studies focused on another topological form of cfDNA, namely the circular cfDNA. Ma *et al.* revealed the existence of a large proportion of mitochondrial DNA (mtDNA) in its original circular form (~16.5 kb) in plasma after digestion of plasma DNA with a restriction enzyme^[49,50]^ [Figure 1, Bottom middle]. The main population of mtDNA derived from the liver was in linear form (91%). In contrast, the majority of mtDNA derived from the hematopoietic system was in circular form (88%). Thus, the topological forms of plasma DNA would be related to the tissue of origin. Sin *et al.* extended the members related to topological forms to extrachromosomal circular DNA (eccDNA)^[29]^. EccDNA molecules could be found in the maternal plasma DNA of pregnant women, but at a relatively low abundance^[29]^. Sin *et al.* further demonstrated that the size distribution of eccDNA molecules is notably different from that of linear cfDNA molecules. EccDNA molecules exhibit two major peaks at 202 bp and 338 bp with a series of sharp 10-bp periodicities^[29]^ [Figure 1, Bottom middle], suggesting that the generation of eccDNA might also involve nucleosomal structures. The peak at 202 bp might be attributed to one nucleosome core and two linkers, while the 338-bp peak might comprise two nucleosome cores and two linkers^[29,51]^. Although there are distinct characteristics between eccDNA and linear plasma DNA, the clearance rates between topological forms did not show a significant difference in the plasma of pregnant women^[51]^. Such an observation seems paradoxical to the previous speculation that the structure of eccDNA might be more stable in plasma^[52]^.

Like linear cfDNA, eccDNA molecules also exhibit tissue-specific properties. Compared with the maternal-derived DNA, the fetal-derived eccDNA appeared to be shorter and hypomethylated. Moreover, eccDNA fragments at the secondary peak cluster (~338 bp) had higher methylation in comparison with the first peak cluster (~202 bp)^[29,51]^. Hence, DNA methylation might play a role in forming the biological properties of eccDNA molecules. Cell-free eccDNA in *Dnase1l3*^−/−^ mice were larger in size than those in WT mice^[30]^. Therefore, DNASE1L3 was suggested to be one of the nucleases involved in digesting eccDNA. Because there were no observable changes in sizes for those eccDNA molecule identified from cellular DNA between WT and *Dnase1l3*^−/−^ mice, DNASE1L3 might play a role in digesting eccDNA extracellularly rather than intracellularly. This finding provided a biological link between nuclease activities and properties of eccDNA in plasma^[30]^ [Figure 1, Bottom middle]. However, eccDNA biogenesis has yet to be fully elaborated. A large percentage of eccDNA molecules contained or were proximal to short direct repeats, suggesting that eccDNA generation might in part involve the microhomology-directed repair^[53]^.

## OTHER TYPES OF LIQUID BIOPSIES

In addition to the intensive studies on cfDNA in plasma, there is growing research interest in urinary cell-free DNA (ucfDNA) molecules that comprise a myriad of important molecular information. One advantage of using ucfDNA is that the urine could be readily sampled repeatedly for surveillance after surgery. Similar to plasma cfDNA, ucfDNA is a mixture containing DNA molecules derived from various tissues, including kidneys, bladders, and blood cells. The tissue origin of ucfDNA could be traced by using its methylation patterns. From the data generated from pregnant women^[54]^ and patients with kidney transplantation^[55]^, two general pathways related to the generation of ucfDNA were elaborated. The first pathway could be cfDNA in blood circulation passing through the glomerular filtration (referred to as transrenal ucfDNA), while the other might be directly contributed by the urinary system (referred to as postrenal ucfDNA).

Owing to the overwhelming activity of DNASE1^[56]^ and the weakened interaction between DNA molecules and histones in the presence of urea, fragmentomic patterns of ucfDNA are distinctive from that in plasma. In contrast to the major size peak of 166 bp in plasma cfDNA, ucfDNA molecules are highly enriched in fragments smaller than 100 bp in size and with enhanced 10-bp periodicities^[54,56-58]^. Chen *et al.* revealed that the DNASE1L3 cutting signature (“C”-end motifs) was relatively diminished in ucfDNA molecules, whereas the DNASE1 cutting signatures (“T”-end motifs) were greatly overrepresented^[56]^. Moreover, a higher jaggedness in ucfDNA molecules was observed when compared with that in plasma cfDNA molecules^[56,59,60]^. Of note, the ucfDNA fragmentation appeared to be time-dependent. For example, the *in vitro* incubation of urine at 37 °C resulted in a constant decrease in ucfDNA concentration, an increase in the amplitude of 10-bp oscillations^[57]^, and an increase in jaggedness^[59]^.

Compared with traditional invasive cystoscopy^[61,62]^, bladder cancer could be detected noninvasively using combined fragmentomic patterns in ucfDNA, with a sensitivity of 93.5% and specificity of 95.8%^[58]^. One previous study showed that serial monitoring of the ucfDNA in renal transplant patients allowed for assessing the transplant allograft status^[58]^. Furthermore, some studies demonstrated that using ucfDNA could more sensitively detect patients with bladder cancer than plasma cfDNA^[63,64]^. These promising findings suggested that the use of ucfDNA could be complementary to the existing plasma cfDNA-based approaches, potentially maximizing the overall diagnostic performance.

Moreover, other types of liquid biopsies, such as cerebrospinal fluid (CSF), have also attracted recent research interest. Mouliere *et al.* demonstrated that the analysis of CSF had improved the detection of patients with brain tumors, compared with either the use of urinary or plasma cfDNA^[65]^. Wu *et al.* studied non-small cell lung cancer patients with leptomeningeal metastases and revealed that 100% of driver mutations were detected in CSF, whereas only 57.8% were detected in paired plasma cfDNA^[66]^. Interestingly, the size shortening of tumor-derived cfDNA was consistently observed in CSF, plasma, and urine samples of glioma patients^[65]^. In addition, the short cfDNA (< 150 bp) in CSF was significantly higher than those in plasma cfDNA^[66]^.

## VIRAL CFDNA MOLECULES

Circulating viral cfDNA molecules have also attracted a lot of recent research interest, showing promise in the detection of cancer types driven by viruses. Using real-time quantitative PCR, Lo *et al.* demonstrated that the detection rate of circulating Epstein-Barr virus (EBV) DNA in plasma was 96% in nasopharyngeal carcinoma (NPC) patients, which was much higher than in controls (7%)^[67]^. Advanced-stage NPC patients had higher EBV DNA quantity than early-stage patients. The results suggested that the EBV cfDNA could serve as a biomarker for NPC. However, because the NPC incidence is very low, the 7% false positivity would lead to a low PPV. Subsequently, the same group adopted a two-time-point testing approach so that a participant with initially positive EBV DNA in plasma at the first test was required to provide another blood sample for a second EBV DNA test within 4 weeks. Only someone determined to be positive at both time points was confirmed as a positive case. Using a prospective cohort of 20,174 participants, Chan *et al.* elucidated that such an approach provided a promising screening tool for NPC among asymptomatic individuals^[68]^, improving the PPV from 3.1% to 11.0% (single-time-point testing), making it possible to identify the majority of NPC patients (75%) at an early stage.

Using massively parallel sequencing targeted to the EBV genome, Lam *et al.* revealed the deferential size profiles of circulating EBV DNA between patients with and without NPC^[69]^. NPC patients generally had a higher amount of circulating EBV DNA, exhibiting higher proportions of longer EBV DNA fragments in plasma than individuals without NPC^[69]^. By leveraging the EBV DNA quantity and EBV DNA size characteristics, the PPV for detecting NPC could be improved to 19.6%, with a 100% sensitivity. Another advantage of the massively parallel sequencing-based test is that it only requires a single blood draw.

Furthermore, it was observed that plasma EBV DNA in NPC patients was hypermethylated. By combining such differential methylation signals, the performance of the NPC test could be further improved, achieving a PPV of 35.1%. These studies indicate that the fragmentomic and epigenetic properties of viral cfDNA are important features for developing sensitive tools for virus-driven cancers. Additionally, Linthorst *et al.* found distinct fragmentation patterns for several DNA viruses compared with human cfDNA, such as adeno-associated virus (AAV), herpes simplex virus (HSV), varicella-zoster virus (VZV), cytomegalovirus (CMV), herpesvirus (HHV), torque teno virus (TTV), hepatitis B virus (HBV), and human papillomavirus (HPV), in maternal plasma DNA of pregnant women^[70]^. However, the biological and clinical implications for viral DNA in pregnancies remained elusive.

## PRE-ANALYTICAL CONSIDERATIONS FOR CFDNA ANALYSIS

Apart from the progress in the field of cfDNA fragmentation, it is still necessary to optimize and standardize the pre-analytical steps during clinical implementation. Plasma DNA was predominantly derived from the hematopoietic system^[71]^. The other solid tissues, such as the liver, placenta, lung, and tumor tissues, generally contribute a small amount of DNA to blood circulation^[10,72-74]^. The released cellular genomic DNA (gDNA) from blood cells into plasma during the delayed blood processing would dilute the signals of interest, such as fetal DNA and tumoral DNA. To minimize blood coagulation and its associated cell death, the EDTA tube is commonly used for blood collection for plasma DNA analysis. However, a previous study demonstrated that gDNA contamination would be introduced into plasma after the blood was stored for more than 6 h before plasma isolation^[75]^. New commercial tube types were developed to control the quality of plasma samples. It was reported that the Streck tube was better than the EDTA tube for preserving plasma samples^[76]^.

Moreover, the fragmentomic profiling of cfDNA molecules is mainly based on double-stranded DNA library preparation. Compared with double-stranded DNA library preparation in short-read sequencing, some studies reported that single-stranded DNA library preparation would be biased toward the ultra-short cfDNA molecules^[25,26]^. Some recently developed methods for enriching ultra-short single-stranded cfDNA demonstrated the potential for cancer detection^[27,77,78]^.

In addition to the analysis of long cfDNA molecules using SMRT sequencing (PacBio), nanopore sequencing (Oxford Nanopore technologies, ONT) could also enable direct, real-time analysis of long cfDNA. Yu *et al.* recently published a study comparing PacBio technology and ONT for cfDNA analysis^[33]^. Notably, both platforms showed the preference to sequence longer DNA fragments, with a stronger bias for PacBio technology. For example, percentages of cfDNA fragments > 500 bp in PacBio were approximately 6-fold higher than in ONT^[33]^. Both PacBio and ONT could generate similar cfDNA end motif profiles, yet displayed platform-dependent patterns. Both platforms achieved a comparable performance of tissue-of-origin analysis based on single-molecule methylation patterns. Although SMRT sequencing generated data with higher percentages of long cfDNA compared with ONT sequencing, a higher absolute number of long cfDNA fragments eligible for the tissue-of-origin analysis were obtained from ONT sequencing because of its much higher sequencing throughput. When analyzing the size and end motif of cfDNA, one should be aware of the analytical characteristics and possible biases of the sequencing platforms being used.

## CONCLUSION

Fragmentation patterns of cfDNA molecules contain a wealth of molecular information related to the tissue of origin. The emerging classes of fragmentomic features, such as long cfDNA molecules, eccDNA, and size modalities around TSS, could accelerate the development of high-performance diagnostic tools for NIPT, cancer detection, monitoring of organ transplantation, and the detection of other diseases such as autoimmune diseases. One advantage of using fragmentomic features is potentially a large number of markers available for differentiating the signals derived from tumor and non-tumor cells at the early stage of cancer, with the ability to localize the tumor. In the NIPT field, the detection of fragmentation aberrations in plasma DNA would expand the diagnostic applications beyond the current focus on chromosomal aberrations. For transplant monitoring, the development of fragmentomic markers would provide an alternative to genetic markers for detecting transplant rejection. The mechanistic understanding of the properties of long cfDNA molecules is still in its infancy. The emerging concept that methylation patterns of long cfDNA molecules serve as intrinsic “molecular barcodes” for tracing the tissues of origin at single-molecule resolution needs to be further elucidated in the various diagnostic applications. Of note, the future standardization of experimental procedures would enhance the overall diagnostic performance when implementing those fragmentation-based approaches in clinical settings.

## DECLARATIONS

### Copyright

© The Author(s) 2022.
